# Unraveling the ECM-Immune Cell Crosstalk in Skin Diseases

**DOI:** 10.3389/fcell.2019.00068

**Published:** 2019-05-07

**Authors:** Oindrila Bhattacharjee, Uttkarsh Ayyangar, Ambika S. Kurbet, Driti Ashok, Srikala Raghavan

**Affiliations:** ^1^School of Chemical and Biotechnology, Sastra University, Thanjavur, India; ^2^Institute for Stem Cell Biology and Regenerative Medicine, GKVK Campus, Bangalore, India

**Keywords:** extracellular matrix, skin diseases, immune cells, crosstalk, therapeutics

## Abstract

The extracellular matrix (ECM) is a complex network of proteins and proteoglycans secreted by keratinocytes, fibroblasts and immune cells. The function of the skin ECM has expanded from being a scaffold that provides structural integrity, to a more dynamic entity that is constantly remodeled to maintain tissue homeostasis. The ECM functions as ligands for cell surface receptors such as integrins, dystroglycans, and toll-like receptors (TLRs) and regulate cellular signaling and immune cell dynamics. The ECM also acts as a sink for growth factors and cytokines, providing critical cues during epithelial morphogenesis. Dysregulation in the organization and deposition of ECMs lead to a plethora of pathophysiological conditions that are exacerbated by aberrant ECM-immune cell interactions. In this review, we focus on the interplay between ECM and immune cells in the context of skin diseases and also discuss state of the art therapies that target the key molecular players involved.

## Introduction

Homeostasis in the skin, the largest organ of the body, is critical for maintaining its structural, protective and regulatory functions. Alterations in this homeostasis, as seen in major skin disorders such as Atopic Dermatitis (AD), Psoriasis, Epidermolysis Bullosa (EB), and skin cancer, lead to impaired skin hydration, thermoregulation, and increased susceptibility to infections (Mellerio, 2010; Romanovsky, 2014; Nyström and Bruckner-Tuderman, 2018). An important role in exacerbating the disease condition is played by dysregulated interactions between two important goal keepers of skin function, the immune cells and the extracellular matrix (ECM). Immune cells play a critical role in the breakdown, synthesis, and remodeling of ECM components that are important for physiological processes such as mammary gland remodeling, lung branching morphogenesis and wound healing (Allori et al., 2008; Rozario and DeSimone, 2010). ECM components such as laminin and collagens, on the other hand, act as ligands for immune cells and facilitate their adhesion and trafficking (Simon and Bromberg, 2017). Proteolytic enzymes secreted by the immune cells generate fragmented laminin and collagen peptides that can serve as chemotactic signals for the recruitment of immune cells (Bonnans et al., 2014). Moreover, ECM components and their physical properties can drive specific activation states of immune cells such as macrophages (McWhorter et al., 2015). In certain chronic disease conditions, excess ECM deposition results in fibrosis that can later develop into cancer (Bonnans et al., 2014). Therefore, maintaining a balance between remodeling by the immune system and ECM dynamics becomes critical to maintain homeostasis.

Skin diseases are largely incurable and therefore remain a major burden; globally posing an adverse socio-economic and psychological impact on the affected populations. The prevalence estimates of psoriasis can go as high as 1% in the U.S. to 8.5% in Norway (Parisi et al., 2013). AD affects up to one-fifth of the population in developed countries (Weidinger and Novak, 2016). Skin cancers, especially non-melanoma skin cancer affects over 3 million Americans yearly with high mortality rates (Apalla et al., 2017). The prevalence of EB has risen to 11.07 per million live births globally and the numbers continue to rise (Fine, 2016). Commonly used treatment regimes for skin diseases include, the use of topical steroids and anti-inflammatory medications to control disease progression (Thomas et al., 2018; Yu et al., 2018). However, there is a paucity of curative therapies, which necessitates further understanding of the mechanisms that exacerbate the disease condition. Thus, focusing on the crosstalk between ECM and immune cells in skin diseases may help identify better targets for therapeutic interventions. In this review, we highlight the bidirectional interactions between ECM and immune cells in the major skin diseases and discuss the therapeutic strategies for dampening this “War of the Titans.”

## The Skin and its ECM

**Figure 1:**
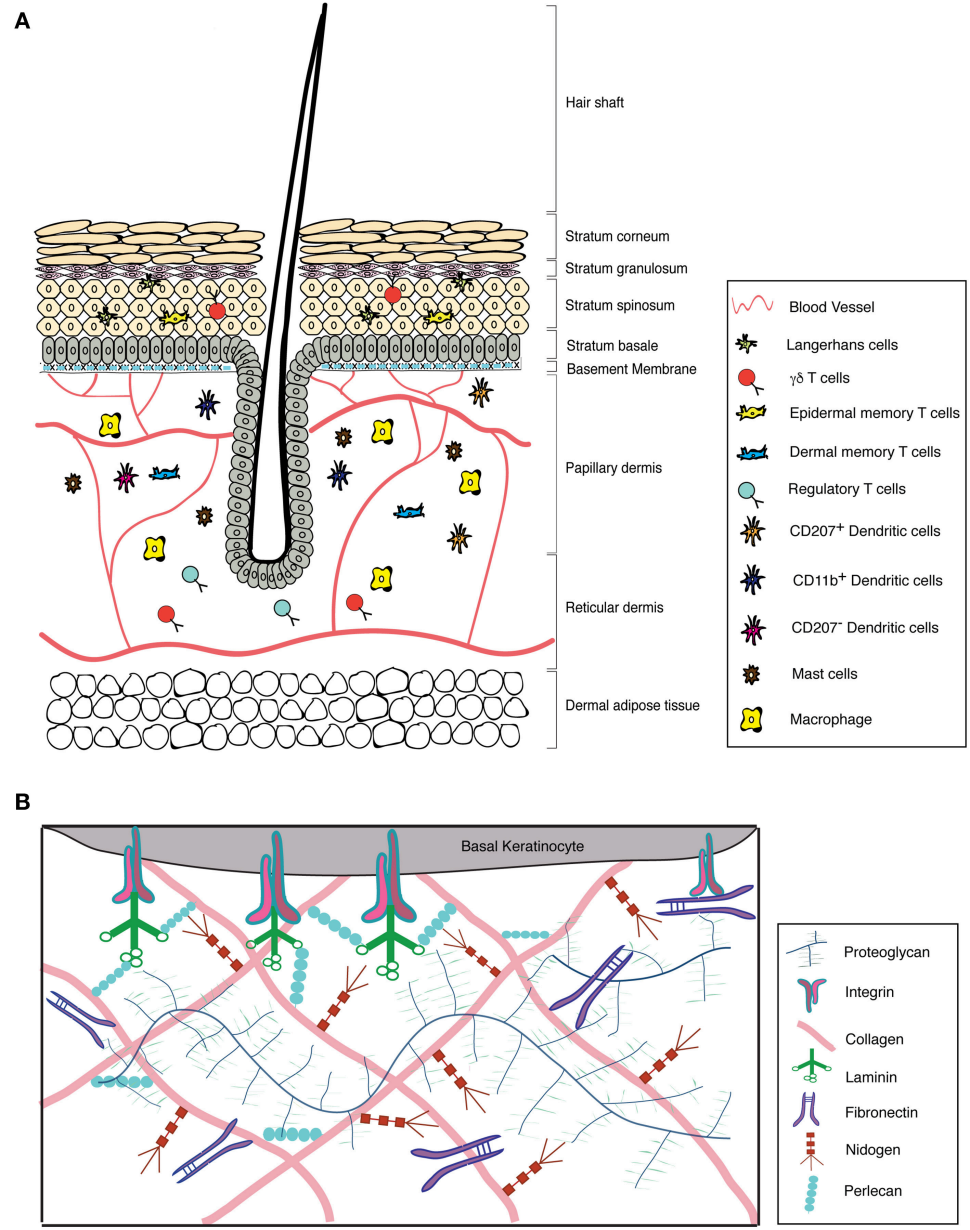
Structure of the skin with its associated ECM and immune cell repertoire. **(A)** Structure of the skin and its immune components. **(B)** Graphical representation of the major components of the skin ECM.

Mammalian skin comprises of a stratified epithelium known as the epidermis and a connective tissue layer termed the dermis. The epidermis is made up of specialized cells containing keratin intermediate filaments known as keratinocytes and is stratified into different layers, namely, basal (stratum basale), suprabasal (stratum spinosum), granular (stratum granulosum), and cornified (stratum corneum) layers (Figure 1) (Fuchs and Raghavan, 2002; Breitkreutz et al., 2013). The basal keratinocytes comprise a pool of proliferating stem cells, which undergo terminal differentiation and eventually lose their nuclei to form the outermost cornified layer (Blanpain and Fuchs, 2006). Each of these epidermal layers is characterized by the expression of different structural proteins. The basal keratinocytes express keratin 5 and 14, which are replaced by keratin 1 and 10 as they differentiate into the spinous layer. The granular layer, as the name suggests, contains keratohyalin granules made up of lipids and precursors of proteins such as loricrin, filaggrin, and involucrin. These proteins and lipids are covalently crosslinked by transglutaminase to form the outermost layer, the stratum corneum that is 10–30 cell layers thick. The stratum corneum is composed of flattened, and enucleated cells embedded in matrix that is rich in proteins and insoluble lipids (Candi et al., 2005). Mutations in any of the above mentioned genes can lead to skin diseases, some of which are discussed later in the review (Chandran, 2013). The dermis is a highly vascularized tissue comprising of fibroblasts, adipocytes and various pools of innate and adaptive immune cells (Mueller et al., 2014). It is divided into a thin upper papillary dermis with loosely arranged collagen fibrils and a thick lower reticular dermis with dense collagen packing. Furthermore, the dermal and hypodermal compartments of the skin also comprise of triglyceride rich adipocyte depot called the dermal white adipose tissue (DWAT) and subcutaneous white adipose tissue (SWAT), respectively (Driskell et al., 2014).

ECM components in the skin are present in the basement membrane and interstitial matrix (Frantz et al., 2010). The BM is a non-cellular array of structural ECM components that separates epidermis from dermis. It is composed of different subtypes of laminins and collagens, primarily laminin 5 (laminin-332) and collagen IV, which are non-covalently connected through perlecan and nidogen (Breitkreutz et al., 2013). This forms a highly dynamic fibrous structure that also functions as a diffusion barrier and serves as a repository of growth factors (Jayadev and Sherwood, 2017). The interstitial matrix is localized to the interstitial spaces in the dermal compartments. These primarily comprise of other collagen subtypes that also impart structural stability to the tissue (Bonnans et al., 2014). Additionally, there are non-structural ECM components called matricellular proteins that are synthesized by the epidermal and dermal cells during development and under non-homeostatic conditions. Matricellular proteins are involved in modulating cell function by interacting with structural ECM proteins as well as with various receptors, hormones and proteases. These interactions are a function of local availability of growth factors and overall composition of the ECM. Tenascin-C (TNC), thrombospondin (TSP), a secreted protein, acidic and rich in cysteine (SPARC), osteopontin (OPN), decorin, periostin (POSTN), and cysteine-rich protein 61 (CCN-1) are some of the major matricellular proteins (Bornstein, 2009). While basement membrane components are relatively stable, matricellular proteins undergo rapid turnover (Bonnans et al., 2014).

Organization of the BM components is mediated through transmembrane heterodimeric cell surface receptors called integrins that are expressed by the basal keratinocytes (Hynes, 2002; Andriani et al., 2003). Integrins are composed of α and β subunits. There are 18 α and 8 β subunits that form 24 different combinations. In the skin epidermis, α2β1, α3β1, and α6β4 are the main integrin pairs that are expressed (Watt and Fujiwara, 2011). Integrin β4 and β1 are components of multi-protein cell-substratum junctional complexes known as hemidesmosomes and focal adhesions, respectively. These structures facilitate the adherence of basal keratinocytes to its underlying BM either via intermediate filaments (in hemidesmosomes) or via actin cytoskeleton network (in focal adhesions) (Tsuruta et al., 2011). ECM-integrin signaling at the BM is indispensable for several cellular processes such as cell proliferation, differentiation, adhesion, migration, and apoptosis (Hegde and Raghavan, 2013). Any disruptions in the ECM-integrin interactions lead to adhesion defects (Nelson and Larsen, 2015). Recent work from our laboratory uncovered the importance of ECM-integrin interaction in embryonic skin homeostasis. We showed that loss of integrin β1 in embryonic skin results in ECM degradation that is exacerbated through a sterile inflammatory response (Kurbet et al., 2016).

## Immunomodulatory functions of ECM

ECM molecules are potent regulators of immune cell function (Table 1). Alterations in the structure and composition of ECM in disease conditions provide immune cells with physical and biochemical cues that, in turn, affect their activation states. Several studies have shown that both enzymatically digested ECM components as well as intact ECM proteins can modulate activation, fate determination, and chemotaxis of immune cells (Lu et al., 2011; Hallmann et al., 2015; Simon and Bromberg, 2017).

**Table 1:** Immune functions of ECM.

**ECM component**	**Immune functions**	**ECM related skin diseases**	**References**
**STRUCTURAL COMPONENTS**			
Laminin	• Laminin affects adhesion, migration, and proliferation of immune cells via integrin signaling. • Fragments or processed peptides of laminin chains act as DAMPs (damage associated molecular patterns), and can affect gene expression of cytokines and MMPs (Matrix metalloproteinases).	JEB and anti-laminin γ1 pemphigoid.	Adair-Kirk and Senior, 2008; Kamata et al., 2013; Senyürek et al., 2014; Simon and Bromberg, 2017
Collagen	• Collagen can bind to immune receptors thereby activating them that, in turn, can inhibit cytotoxic functions of natural killer and T cells. Collagen can affect proliferation of other immune cells. • Collagen can interact with other immune activating matricellular proteins such as cochlin to mount anti-pathogenic responses. • Collagen fragments can increase secretion of IL-1β by blood monocytes.	RDEB (Recessive dystrophic epidermolysis bullosa), EBA (Epidermolysis bullosa aquisita), Systemic lupus erythematosus and scleroderma.	Jimenez et al., 1996; Meyaard et al., 1997; Stultz et al., 2007; Rygiel et al., 2011; An and Brodsky, 2016; Nyström et al., 2018
Elastin	• Elastin peptides act as chemotactic agents for immune cells.	Cutis laxa, pseudoxanthonum elasticum	Uitto and Shamban, 1987; Fulop et al., 2012
ECM-1	• ECM-1 suppresses Th17 responses and can be secreted by Th2 immune cells in autoimmune diseases. • ECM-1 can interact with αv integrins and block TGF-β activation.	Lipoid proteinosis, lichen sclerosus	Oyama et al., 2003; Su et al., 2016
Fibronectin	• Fibronectin variants obtained by splicing and partial folding can activate TLR signaling and induce secretion of cytokines and chemokines by fibroblasts. • Fibronectin can be produced by Th1 cells and may be important in opsonizing intracellular pathogens.	Atopic dermatitis, psoriasis, perforating skin disorders.	Sandig et al., 2009; McFadden et al., 2011; Kelsh et al., 2014
**MATRICELLULAR COMPONENTS**			
Tenascin C	• Tenascin affects adhesion properties of monocytes, B and T cells. • Tenascin C binds to TLR-2 & TLR-4 and initiates a pro-inflammatory cascade.	Basal cell carcinoma, psoriasis, solar keratosis, Bowen's diseases	Rüegg et al., 1989; Schalkwijk et al., 1991; Zuliani-Alvarez et al., 2017
Osteopontin	• OPN promotes Th17 and Th1 while suppressing Th2 responses.	Systemic lupus erythematosus, psoriasis, contact dermatitis	Mori et al., 2008; Buback et al., 2009; Reduta et al., 2015
CCN	• CCN helps in macrophage adhesion via binding to cognate integrin receptors. • CCN activates NFκB mediated transcription followed by a pro-inflammatory cascade. • Promotes IL1β secretion by psoriatic keratinocytes.	Psoriasis, scleroderma	Bai et al., 2010; Sun et al., 2015; Henrot et al., 2018
Decorin	• Mounts pro-inflammatory responses by binding to TLR-2 and -4 or by suppressing TGF-β responses.	Delayed type hypersensitivity	Yamaguchi et al., 1990; Seidler et al., 2011; Bocian et al., 2013; Frey et al., 2013
TSP-1	• Promotes anti-tumor M1 macrophage recruitment via release of reactive oxygen species. • Facilitates adhesion of neutrophils and serves as chemotactic agent for monocytes and neutrophils. • Activates TGF-β	N/A	Bornstein, 2001; Martin-Manso et al., 2008
Periostin (POSTN)	• Activates keratinocytes by binding to αv integrins and stimulates them to secrete TSLP that can trigger a Th2 response.	Atopic dermatitis, dermal fibrosis	Liu et al., 2014; Yamaguchi, 2014

## Skin Immune Cell Repertoire and its Role in Maintaining ECM Dynamics

The skin contains a diverse population of immune cells that continuously surveil the organ to protect from assaults and maintain homeostasis. The epidermal and dermal compartments of the skin have distinct populations of immune cells that perform multiple functions including antigen cross-presentation to effector cells, tolerance induction, mediation of inflammation, promotion of growth and repair, regulation of stem cell quiescence, and maintenance of epidermal differentiation (Di Meglio et al., 2011). Langerhan cells and dendritic epidermal T cells are primarily located in the epidermis while, distinct populations of other dendritic cells and macrophages are present in the dermis (Heath and Carbone, 2013). Mast cells and T cell populations including αβ, γδT, Tregs, Th17, and memory T cells get recruited to both the epidermis and dermis depending on the homeostatic status of the skin (Matejuk, 2018). Details of the expression markers, location, and regulatory roles of immune cells in maintaining skin homeostasis are listed in Table 2.

**Table 2:** Immune cell repertoire in skin.

**Immune cell**	**Subsets**	**Marker expression**		**Localization**	**Functions in skin**	**References**
		**Human**	**Mouse**			
**Dendritic cells (CD11c**^**+**^**, MHC-ll**^**+**^**)**	Epidermal dendritic cells					
	Langerhans cells	CD1a^+^CD80,CD86,HLA-DR^+^CD83DC-LAMP	CD207^+^ (Langerin), CD11b^+^, CD103^−^, CD86^+^, EpCAM^+^, SIRPa^+^	Epidermis, ORS of hair follicle	• Antigen cross-presentation. • Th17 cell differentiation. • CD4+ follicular helper T cell and Th2 cell proliferation. • IgE production during allergic conditions. • Tolerance induction. • Activation of skin resident regulatory cells. • Mediation of inflammation.	Hoeffel et al., 2012; Singh et al., 2016; Kaplan, 2017
	Dermal dendritic cells					
	1. Langerin+ DC	CD141^hi^, CD14^+^	CD45^+^, CD11b^lo^, CD11c, MHC-II^+^, CD103^+^, XCR1^+^, EpCam^−^, Sirpa^−^	Dermis	• Phagocytosis of cell debris and infectious microorganism. • Activation of naïve T cells through antigen presentation. • Regulation of Th1 responses. • Promotion of cytotoxic and memory responses of CD8+ T cells.	Nagao et al., 2009; Henri et al., 2010; Chu et al., 2011; Flacher et al., 2014; Mildner and Jung, 2014
	2. CD11B^+^ DC	CD1a^+^, CD1c^+^	CD45^+^, CD11b^++^, CD11c, MHC class II, CD205^+^, CD103 -EpCam^−^, Sirpa^+^	Dermis	• Clearance of infection.	
	3. Langerin-XCR1^−^ DC	ND	CD207^−^, CD11b^−^, EpCAM^−^, Sirpa^+^, XCR1^−^, CX3CD1^++^	Dermis	ND	
**Macrophage**		CD11b, F4/80, CD163, factor XIIIa, CD16, CD32 and CD64	CD45^+^, MHC II^+^, MERTK^+^, CCR^lo^, F4/80, CD64^+^	Dermis	• Phagocytosis. • Modulation of tissue environment. • Promotion and suppression of inflammation. • Extravasation of neutrophils during inflammation. • Regulation of hair bulge stem cell populations.	Malissen et al., 2014; Yanez et al., 2017
**T cells CD3**^**+**^	Dendritic epidermal T cells CD8^+^, Vγ3Vδ1 TCR			Epidermis and ORS of hair follicle	• Regulation of antimicrobial function. • Prevention of tumor formation. • Promotion of wound closure. • Maintenance of keratinocyte homeostasis through production of growth factors.	Macleod et al., 2013
	Memory T cells (Trm) (CD8^+^, CD103^+^)					
	1. Epidermal Trm	CD45RO^+^,CLA^+^,CCR4^+^	CD49^+^, CD49	Epidermis	• Antimicrobial defense.	Clark et al., 2006; Zaid et al., 2014; Cheuk et al., 2017
	2. Dermal Trm		CD49^−^, CD4^+^	Dermis	• Antimicrobial defense.	
	γδT cells (DETC)	CD8^+^, γδ TCR^+^	CD8^+^, γδ TCR^+^	Epidermis and dermis	• Promotion of Hair follicle regeneration. • Extravasation of neutrophils during inflammation. • Synthesis of keratinocytes and fibroblast growth factors.	Bos et al., 1990; Sumaria et al., 2011; Gay et al., 2013; Cruz et al., 2018
	αβT cells	CD8^+^/CD4^+^,αβ TCR ^+^	CD8^+^/CD4^+^, αβTCR ^+^	Epidermis and dermis	• Cytokine synthesis during tissue damage and infection. • Regulation of hair cycle. • Recruitment of other immune cells. • Promoting cell cytotoxicity.	
	Regulatory T cells (Tregs)	FOXP3^+^, CD45RO^+^,CD4^+^,CD25^+^,CD127^−^	FOXP3^+^, CD25^+^	Around hair follicles	• Epithelial stem cell differentiation. • IL-17 secretion in inflammatory condition. • Maintenance of self-tolerance. • Suppression of inflammation.	Clark, 2010; Sakaguchi et al., 2010; Ali et al., 2017
	Th17 cells	CD4^+^,RORC^+^,CD161^+^	CD4^+^, CD161^+^, CCR6^+^, IL17A^+^, RORyt^+^	Epidermis and dermis	• Protection against pathogens by inducing synthesis of antimicrobial peptides. • Promotes synthesis of inflammatory cytokines and growth factors.	Koga et al., 2008; Maggi et al., 2010; Peck and Mellins, 2010
**Mast cells**		FcyRI^−^, FcyRIIa,CD30	CD117^+^, CD25^+^	Epidermis and dermis	• Pathogens, production of antimicrobial peptides. • Regulation of allergic responses. • Maintenance of epidermal differentiation.	Sehra et al., 2016

Immune cells are one of the most important regulators of ECM degradation, synthesis, assembly and remodeling. Mechanisms by which immune cells accomplish this include–(i) synthesis of enzymes that remodel ECM components, (ii) synthesis of cytokines and growth factors that induce ECM synthesis or degradation, and (iii) synthesis of ECM components.

- Synthesis of enzymes that remodel ECM components: Metalloproteinases are enzymes secreted by the immune cells that cleave ECM proteins leading to alterations in the physical and biochemical properties of the tissue. Degradation of ECM by metalloproteinases aids in the trafficking of immune cells, generates bioactive peptides, and releases the sequestered growth factors (Mott and Werb, 2004). Metalloproteinases include MMPs, (matrix metalloproteinases) adamlysins including ADAMS (a disintegrin and metalloproteinases), ADAMTs (ADAMs containing a thrombospondin motif), Meprins as well as serine proteases such as plasmins, granzymes, and cathepsins. These enzymes cleave collagens (MMP-1, -2, -3, -7, -8, -9, -10, -11, -12, -13, -14, -16, -19, -25, -26; ADAM-10, -12 and -15; Meprin a and b), elastins (MMP-2, -3, -7, -9, -10, -11, - 12), gelatins (MMP-2, -3, -7, -9, -12, -14, -15, -16, -17, -23A, - 23B, -24, -25; ADAM-9, -10, -12, -15), aggrecans (MMP-3, -13; ADAMTs), and laminins (MMP-3, -7, -11, -12, -14, -15, -19, Meprin α and β) (Bonnans et al., 2014). Several studies have highlighted the important roles played by metalloproteinases, derived from immune cells, in remodeling the ECM thereby driving the pathophysiology and severity of various diseases. The T cell repertoire, particularly Th1 cells secrete gelatinases, MMP-2 and -9 that further stimulate the release of gelatinases from macrophages (Oviedo-Orta et al., 2008). In squamous cell carcinoma, mast cells, macrophages and neutrophils secrete MMP-9 that exacerbates the disease condition by increasing keratinocyte hyperproliferation and metastasis (Coussens et al., 2000). Dendritic and Langerhans cells in skin secrete MMP-2 and -9 to facilitate their trans-epithelial migration for antigen presentation (Ratzinger et al., 2002). Monocytes and lung resident macrophages are the primary source of MMP-8 that eventually contributes to fibrosis in idiopathic pulmonary fibrosis (Craig et al., 2014). Likewise, MMP-8 and -12 is synthesized by neutrophils and macrophages in cystic fibrosis accelerating disease progression (Wagner et al., 2016). Increased expression of MMP-9 by splenic and infiltrating T cells correlates with the growth in mammary tumors (Owen et al., 2003). Furthermore, in UVB irradiated skin, neutrophil elastase causes the degradation of ECM components by activating MMP-1, and MMP-2 (Takeuchi et al., 2010).
- Synthesis of cytokines and growth factors that induce ECM synthesis or degradation: Cytokines (IL-4, IL-13, and IL-33) and growth factors (TGF-β) secreted by immune cells stimulate the synthesis of ECM components (Verrecchia et al., 2001; Fujitsu et al., 2003; Rankin et al., 2010). In mouse dermal fibroblasts, TGF-β signaling results in the activation of SMAD2/3, which, in turn, increases the expression of ECM, related genes such as *Col1a1* and *Col3a1* (Verrecchia et al., 2001; Li et al., 2003). IL-13 produced by innate lymphoid cells stimulates the differentiation of fibroblasts to myofibroblasts increasing collagen synthesis and its accumulation in fibrosis (Fichtner-Feigl et al., 2006). In bronchial asthma, Th2 cytokines IL-4 and IL-13 stimulate the synthesis of periostin from bronchial fibrocytes, leading to sub-epithelial fibrosis (Takayama et al., 2006; Aoudjehane et al., 2008). On the other hand, IL-6 and TNF-α produced by inflammatory immune cells can reduce the synthesis of MMP-2, which, in turn, protects liver hepatocytes from fibrosis (Bansal et al., 2005).
- Synthesis of ECM components: Immune cells can themselves be a source for various ECM proteins. Macrophages and T cells present in tuberculosis associated granulomas produce OPN that aid in their chemotaxis, adhesion, and proliferation (O'regan et al., 1999). In a subset of patients with systemic stenosis, circulating CD14^+^ monocytes overexpress versican, which is associated with the aggressive fibrosis (Masuda et al., 2013). TAMs (tumor associated åmacrophages) modify the architecture of the tumor matrix by synthesizing proteoglycans, fibronectin, OPN, SPARC and various collagen subtypes (Liguori et al., 2011).

Taken together, these studies point to a critical role played by immune cells in modulating ECM dynamics during normal development and in disease states.

## Skin Diseases

### Atopic Dermatitis

Atopic dermatitis (AD) is a chronic, relapsing and TH2 cell/IgE driven inflammatory skin disorder characterized by intense pruritus and eczematous lesions (Bieber, 2008). The onset of AD is caused by barrier dysfunction, due to heritable mutations in the filaggrin (*FLG*) gene, and environmental triggers culminating in loss of skin hydration, enhanced sensitization to allergens and susceptibility to pathogenic agents (Weidinger and Novak, 2016). Clinical manifestations of AD occur both in children (Childhood AD) and adults (Adult AD) with similar clinical symptoms but with discrete pathophysiologies. While childhood AD is characterized by additional Th17/Th22 driven cascades along with tight junction and lipid metabolism disorders, AD in adults is associated with a Th1 specific response with aberrations in the epidermal differentiation complex (Brunner et al., 2018). AD usually precedes other allergic disorders like food allergies, allergic rhinitis, and asthma, commonly referred to as the “Atopic March,” the mechanism for which remains undefined (Dharmage et al., 2014).

Abnormalities in BM composition, suggesting ECM dysfunction, have been reported in AD. Affected skin in AD patients has decreased BM thickness, along with reduced expression of collagen-IV and integrin α6 in the BM zone (Kim et al., 2018). A report by Shin et al. (2015) also suggested that the loss of the regenerative capability of the skin correlated with lower expression of p63 protein, a putative stem cell marker, in AD affected skin (Shin et al., 2015). Several SNPs (single nucleotide polymorphisms) in *LAMA3* gene correlated with increased susceptibility to AD (Stemmler et al., 2014). In acute AD, an increase in the expression of hyaluronan (HA), an extracellular polysaccharide, and hyaluronan synthase 3 (HAS3), an epidermal specific enzyme responsible for the synthesis and extracellular transport of hyaluronan, is observed (Ohtani et al., 2009). Increased expression of HA has been associated with abnormal keratinocyte differentiation, a hallmark of AD (Malaisse et al., 2014).

IL-4 and IL-13 are important cytokines known to play a critical role in AD pathogenesis. *In situ* hybridization studies on skin biopsy samples show a greater number of IL-13 positive cells in asymptomatic, acute and chronically affected AD patients compared to unaffected individuals (Hamid et al., 1996). Optimum expression of IL-13 is critical to maintain epidermal barrier homeostasis since both excess and insufficient levels of IL-13 provoke epidermal barrier dysfunction (Strid et al., 2016). IL-4 and IL-13 downregulate the expression of filaggrin, involucrin, loricrin, and the production of antimicrobial peptides. This exacerbates the skin barrier dysfunction and predisposes AD-affected skin to infection by microbes (Howell et al., 2007; Kim et al., 2008; Kisich et al., 2008). IL-4 is also shown to repress the expression of fibronectin in immortalized human keratinocytes (Serezani et al., 2017). In a human skin equivalent model system, IL-13 expression leads to the loss of BM structure and regenerative capability of the skin, recapitulating the AD phenotype (Shin et al., 2015). Furthermore, IL-13 treated human keratinocytes attract CD4^+^ CCR4^+^ T cells *in vitro*, providing a mechanism for a feed-forward loop that recruits more Th2 cells to acute AD lesions (Purwar et al., 2006). A critical correlation exists between IL-13 and MMP-9 mediated BM protein remodeling in AD. IL-13 is co-expressed with MMP-9 in skin samples from atopic eczema patients and it also increases MMP-9 synthesis in cultured keratinocytes (Purwar et al., 2008). IL-4 and IL-13 treated keratinocytes show an overexpression of TNC aggravating the inflammatory response (Ogawa et al., 2005). While mast cells and CD4^+^/CD8^+^ T cells are well known sources of IL-13 in AD affected skin, other immune cells including macrophages and dendritic cells can also be potential sources, which warrants further investigation (Obara et al., 2002). Taken together, these studies highlight the critical role played by IL-4 and IL-13 in driving AD pathogenesis.

Immune cell mediated remodeling of ECM in AD has been widely reported. BM remodeling matrix metalloproteinases (MMPs) including MMP-1, -3, -7, -8, and -9 is involved in AD pathophysiology (Harper et al., 2010). AD patients have enhanced serum levels of MMP-8 and -9 (Devillers et al., 2007). An immediate early effect in increase of MMP-9 by langerhans cells is observed upon treatment of skin with hapten, a well-known model for contact hypersensitivity (Kobayashi, 1997). The hyperplasic epidermis in AD secretes thymic stromal lymphopoeitin (TSLP), an IL-2 family cytokine, that activate DCs. DCs, in turn, produce MMP-3 and -9 that aid in their migration to lymph nodes where they activate naïve T cells (Ratzinger et al., 2002). Additionally, degranulation product of epidermal mast cells in AD-affected skin is associated with increased expression of MMP-7 (Landriscina et al., 2015). These reports indicate an indirect immune cell mediated BM remodeling in the context of AD. However, focused studies addressing this crosstalk are limited.

Monocytes and macrophages also play an important role in AD pathogenesis. An insightful review on the role of macrophages in AD has been previously published (Kasraie and Werfel, 2013). In AD skin, the epidermis overexpresses monocyte chemoattractant protein 1 (MCP-1) and, this results in the recruitment of monocytes in a CCR-2 dependent manner (Vestergaard et al., 2004). Acute and chronically inflamed AD skin show enhanced infiltration of heterogeneous pools of macrophages (Kiekens et al., 2001). Immunohistochemistry and ELISA analysis of AD skin and peripheral blood show increased CD163 expressing macrophages. Their role in aggravating disease condition is suggested by *in vitro* studies where the macrophages show an impaired TLR-2 signaling and increased secretion of pro-inflammatory cytokines such as IL-1β, IL-8, and IL-6 (Niebuhr et al., 2009). Given the preponderance of evidence on ECM-macrophage crosstalk in diseases, it is tempting to speculate that these heterogeneous pools of macrophages may play an important role in ECM remodeling.

Interestingly, recent studies point to the importance of ECM driven cascades in AD pathogenesis. Mitamura et al., showed that IL-13 upregulates the synthesis of POSTN which, in turn, increases IL-24 expression in the epidermal keratinocytes. IL-24, in turn, downregulates the expression of filaggrin, that contributes to the barrier dysfunction seen in atopic dermatitis (Mitamura et al., 2018). Another study showed that, the binding of TNF superfamily member 14 (TNFSF-14) to its receptor HVEM (herpes virus entry mediator) on keratinocytes resulted in the synthesis of POSTN and development of AD. Blocking this pathway by HVEM deletion and HVEM specific antibody resulted in suppression in AD symptoms (Herro et al., 2018). The importance of OPN in AD is highlighted in several studies. Its expression is elevated in the serum of patients with allergic contact dermatitis (Reduta et al., 2015). In disease conditions, immune cells such as T cells, NKT cells, and plasmacytoid dendritic cells synthesize OPN that acts as a bridging molecule between the innate and adaptive immune system (Clemente et al., 2016). OPN promotes a pro-inflammatory environment by enhancing production of IL-12 by macrophages and suppressing IL-27 production from conventional dendritic cells, thereby activating Th1 and Th17 cells (Clemente et al., 2016). OPN binds to CD44 receptors on mast cells leading to its recruitment and degranulation (Bulfone-Paus and Paus, 2008). Thus, studies on matricellular proteins POSTN and OPN have highlighted therapeutically targetable pathways that are the key drivers of AD symptoms.

Collectively, AD is characterized by a multitude of dysregulated immune responses, as well as close bidirectional interactions with matricellular and basement membrane components that exacerbate disease pathophysiology.

### Psoriasis

Psoriasis is a skin disorder associated with pathological changes such as appearance of erythematous lesions, scaling of the epidermis, inflammation, and vascular abnormalities (Nestle et al., 2009). Lesions appear as red and scaly plaques in areas like the elbow, joints, lower back, and the scalp (Boehncke and Schön, 2015). It is classified into plaque, guttate, erythrodermic, and pustular types with plaque psoriasis (or psoriasis vulgaris) being the most common type associated with about 90% of the cases (Dubois Declercq and Pouliot, 2013). Based on the age of onset of the disease, psoriasis is classified as Type I (occurs in individuals below 40 years of age) and Type II (sets in after 40 years of age) (Langley et al., 2005). Type I is more common, runs in families and is associated with more severe phenotypes compared to Type II (Schmitt-Egenolf et al., 1991). The exact cause of this disease is still unknown. However, a combination of genetic and environmental factors such as infection, injury, and various administered drugs may be responsible for disease onset (Langley et al., 2005).

Cell death due to injury or trauma is thought to trigger the keratinocytes to produce antimicrobial peptides like LL-37 that activate plasmacytoid dendritic cells (pDCs) (Morizane and Gallo, 2012). The pDCs secrete IL-12 and IL-23 and mount a T cell mediated inflammatory response. The T cells, primarily Th1, Th17, and Th22, secrete the cytokines IFN-γ and TNF that promote inflammation in psoriatic skin. An increase in the infiltration of neutrophils, dendritic cells and T cells in the lesional skin of psoriatic patients evokes a crosstalk between the activated keratinocytes and immune cells (Baliwag et al., 2015; Albanesi et al., 2018). Although, T cells and dendritic cells primarily drive psoriasis, interesting roles for macrophages and neutrophils have recently emerged. Depletion of macrophages by clodronate treatment leads to alleviation of the psoriatic phenotypes in mice (Stratis et al., 2006). Neutrophil derived cytokines such as IL-1β, IL-6, IL-17, and IL-23 are associated with specific gene expression changes in keratinocytes that result in epidermal hyperproliferation and abnormal differentiation (Terui et al., 2000; Tecchio et al., 2014). In contrast, antibody mediated neutralization of neutrophils does not reduce inflammation. Interestingly, neutrophils in psoriatic skin form web-like extracellular traps, referred to as NET (Neutrophil extracellular trap) that is composed of chromatin and antimicrobial components such as LL-37, neutrophil elastase, and myeloperoxidase (Fuchs, 2007). The formation of NET is associated with NETosis, which involves killing of pathogens through the release of IL-17 and antimicrobial peptides (Hu et al., 2016).

Several studies have underscored the contribution of ECM proteins in the pathogenesis of psoriasis. A disorganized BM (laminin) in the psoriatic skin is associated with keratinocyte-derived plasminogen activating factor (McFadden et al., 2012). Laminin-332 and -511 are overexpressed in the BM of psoriatic skin lesions. Furthermore increased expression of laminin-511 in HaCat cells stimulated proliferation and inhibited apoptosis providing a mechanism for the observed epidermal hyperplasia (Natsumi et al., 2018). Additionally, alteration in laminin α1 chain along with overexpression of fibronectin in the papillary dermis and T cell lymphokines are correlated with abnormal cellular morphology (Vaccaro et al., 2002). While laminins are shown to facilitate neutrophil recruitment and enhance the phagocytic ability of dendritic cells, its role in immunomodulation in the context of psoriasis remains elusive (Wondimu et al., 2004; García-Nieto et al., 2010; Simon and Bromberg, 2017).

CCN-1, a matricellular protein is overexpressed in the lesional and non-lesional psoriatic epidermis in response to pro-inflammatory cytokines IL-17 and TNF-α. It binds to integrin α6β1 on keratinocytes and activates PI3K/Akt/NF-κB pathway and result in the upregulation of *ICAM, HLA-DR, and HLA-ABC* genes. These genes, in turn, increase the immune cell-keratinocyte interaction and mount an inflammatory response. Its role in promoting epidermal hyperplasia and inflammation was further demonstrated in IL-23 induced psoriatic skin lesions, where the knockdown of CCN-1 ameliorated the phenotypes (Sun et al., 2015). Other studies have shown that CCN-1 stimulates secretion of the cytokines IL-1β and IL-8 by psoriatic keratinocytes via the p38 MAPK and JNK/NF-κB signaling pathways, respectively underscoring its role in regulation of inflammation (Sun et al., 2017; Wu et al., 2017). Furthermore, CCN-1 promotes CCL-20 chemokine synthesis from hyperplasic epidermis of psoriatic skin that causes increased recruitment of immune cells (Li H. et al., 2017). Taken together, these studies highlight an important role played by CCN-1 in regulation inflammation in psoriatic skin.

OPN, expressed by epithelial cells, is shown to act as a potent pro-inflammatory stimulant and activates immune cells such as T-cells, dendritic cells, and macrophages/monocytes (Clemente et al., 2016). Interaction of OPN with integrin and CD44 leads to the promotion of Th1 response and suppression of Th2 response. In psoriasis, OPN is highly expressed in the PBMCs (peripheral blood mononuclear cell), skin and plasma. Buommino et al showed that OPN secreted by PBMCs enhances the expression pro-inflammatory cytokines such as IL-1β, TNF-α, and IFN-γ. TNF-α upregulates OPN expression while anti-TNF-α antibody treatment abrogates its synthesis (Buommino et al., 2012).

Overall, these studies suggest that ECM components play a critical role in psoriatic skin by acting as co-stimulatory factors for keratinocytes and immune cell activation leading to chronic progression of the disease.

### Epidermolysis Bullosa

**Figure 2:**
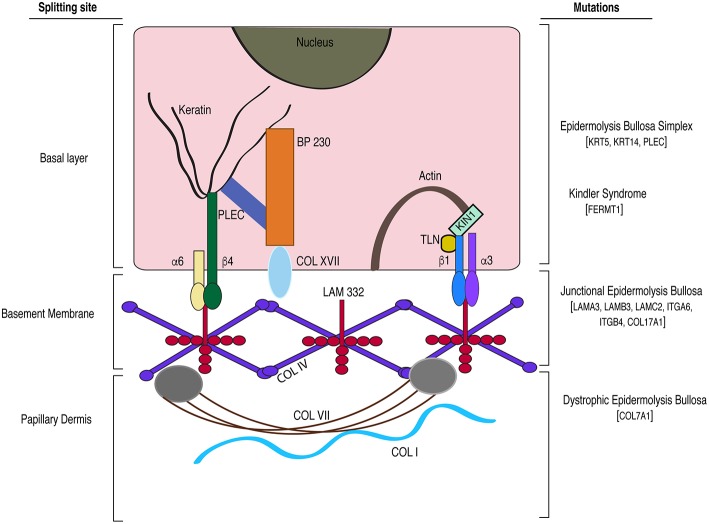
A model depicting the gene mutations and the site of splitting associated with the different subtypes of epidermolysis bullosa. BP, bullous pemphigoid; TLN, Talin; α6, integrin alpha 6; β4, integrin beta 4; α3, integrin alpha 3; β1, integrin beta 1; LAM 332, laminin-332 protein; PLEC, plectin; COL, collagen; KIN1 (FERMT1), kindlin-1; KRT, keratin; ITG, integrin.

Epidermolysis bullosa (EB) is a group of rare heritable diseases characterized by blistering and fragile skin. It is associated with anomalies such as palmar-plantar thickening, syndactyly, dysphagia, gingival hyperkeratosis, cardiomyopathy, and osteoporosis (Maldonado-Colin et al., 2017). The blistering results from mechanical stress that eventually gives rise to chronic wound conditions (Medeiros and Riet-Correa, 2015). EB is caused by mutations in hemidesmosomal and extracellular matrix genes and the mode of inheritance can be either dominant or recessive. EB has been broadly categorized into simplex (EBS), junctional (JEB), dystrophic (DEB) types, and kindler syndrome (Shinkuma, 2015; Barna et al., 2017). The severity of disease is a function of the location of the split and the genes affected. So far mutations in at least 19 genes are reported to cause different types of EBs (Vahidnezhad et al., 2018). The site of splitting associated with various mutations is illustrated in Figure 2.

**EBS** is caused by mutations in the genes coding for either keratin 5 (*KRT5*) or keratin 14 (*KRT14*) accounting for the four major subtypes. *KRT5/14* form a stable cytoskeletal network and are involved in maintaining tissue integrity (Coulombe and Lee, 2012). Mutations in *KRT5/14* result in rupture of basal keratinocytes leading to increased susceptibility to infection and dehydration. The least severe form of EBS is Weber-Cockayne type characterized by mild blistering restricted to feet and hands whereas the most severe form known as Dowling-Maera type is marked by extensive blistering, which however seems to get better with age. The other two subtypes are Koebner type and EBS with mottled pigmentation (Chung and Uitto, 2010a). Additionally, mutation in the plectin (*PLEC*) gene, a hemidesmosomal protein, is associated with a rare type of EBS known as Ogna type (Pfendner et al., 2005). The features common to all the subtypes are thickened palm and soles (palmoplantar keratoderma), non-scarring blisters, alopecia and dystrophic nails (Chung and Uitto, 2010a). EBS is the most prevalent and mildest type of EB, and exhibits an autosomal dominant inheritance pattern (Coulombe et al., 2009). Mouse knockouts for *Krt5* and *Krt14* serve as good models for studying EBS disease pathogenesis (Coulombe et al., 1991; Peters et al., 2001).

**JEB** is an autosomal recessive form of EB caused by mutations in the laminin-332 chains encoded by *LAMA3, LAMB3, LAMC2* genes, and *COL17A1* genes that code for collagen XVII/BP180 protein (Sawamura et al., 2010). Collagen-XVII is an important structural element of the hemidesmosomal complex that together with laminins in the BM, maintain the dermal-epidermal adhesion. JEB can be further classified into two subtypes; Herlitz and non-Herlitz based on its severity. Patients with the Non-Herlitz subtype display blisters primarily in the feet, elbows and knees, which are non-life threatening (Yancey and Hintner, 2010). The Herlitz subtype, on the other hand, causes severe disease, and is associated with widespread blistering of the lining of oral and digestive tracts, syndactyly and alopecia, and restricts life expectancy to 1 year after birth (Yuen et al., 2011).

*Col17a1* knockout mice display the blistering pattern associated with the non-Herlitz subtype and show prolonged survival; whereas *Lama3* and *Lamc2* knockouts recapitulate the Herlitz type of EB, exhibiting severe skin blistering resulting in perinatal lethality (Bubier et al., 2010; Natsuga et al., 2010). Two other rare autosomal recessive forms of EB include EB with pyloric atresia (EB-PA) and EB with muscular dystrophy (EB-MD). EB-PA is caused by mutations in *ITGA6* and *ITGB4* genes whereas EB-MD is associated with mutations in the *PLEC* gene (Shimizu et al., 1999; Yancey and Hintner, 2010). *Itga6* and *Itgb4* knockout mouse models recapitulate the dermal-epidermal detachment leading to development of EB-PA (Georges-Labouesse et al., 1996; Mencía et al., 2016).

**DEB** is associated with mutations in *Col7a1* gene resulting in the loss of collagen-VII protein. Collagen-VII is an anchoring fibril protein crucial for dermal-epidermal integrity and is present in the BM on the side of papillary dermis (Chung and Uitto, 2010b). The two major subtypes of DEB based on mode of inheritance and severity include autosomal dominant (DDEB) and recessive (RDEB) types. Unlike EBS and JEB, DEB is associated with extensive scarring (Shinkuma, 2015). RDEB, the more severe subtype is characterized by pseudosyndactyly, alopecia with scarring, and esophageal stenosis that leads to dysphagia. This disease is further complicated by the development of aggressive squamous cell carcinoma in juvenile patients (Shinkuma, 2015). The conditional *Col7a1* KO mice recapitulate all of the phenotypes of RDEB and has proven to be an invaluable tool to understand disease progression as well as to test therapeutic interventions (Natsuga et al., 2010).

**Kindler syndrome** is the only skin disease known to be associated with actin cytoskeleton related abnormalities and is characterized by variable planes of cleavage in the skin (Medeiros and Riet-Correa, 2015). Keratinocytes express kindlin-1, a protein that regulates cellular processes such as proliferation, migration and cell-substratum adhesion (Rognoni et al., 2016). Mutations in the *FERMT1* gene, coding for the kindlin-1 protein, lead to this rare fragile skin blistering disorder with symptoms similar to other EB subtypes (Lai-Cheong et al., 2009). Kindlin-1 deficient mice display skin atrophy without blister formation (Ussar et al., 2008). The loss of kindlin-1 from keratinocytes results in dysregulated integrin signaling and focal adhesion turnover (Margadant et al., 2013).

Another form of EB called aquisita **(EBA)** is caused by the production of autoantibodies against collagen-VII protein. The two subtypes of EBA are classical and the inflammatory types. The classical form is characterized by trauma induced blistering in adults whereas the inflammatory form starts in childhood and show more widespread blistering that is not restricted to the injured sites (Kasperkiewicz et al., 2016).

The null and conditional KO mouse models that have been discussed above recapitulate various aspects of disease progression of the EB class of skin disorders and are being used as platforms to develop therapeutic interventions for these devastating diseases (Georges-Labouesse et al., 1996; Cao et al., 2001; Peters et al., 2001; Bruckner-Tuderman et al., 2010).

Insights into the role of inflammation and immune cells in EB pathophysiology have come from the studies on *Krt*5, *Itgb4*, and *Col7a1* knockouts that cause EBS, EB-PA, and DEB, respectively. Increased expression of chemokines and cytokines such as CCL-2, CCL-19, CCL-20, IL-6, and IL-1β is reported in *Krt*5 knockout skin. The cytokine expression is associated with a 2-fold increase in the recruitment of Langerhans cells to the EB affected skin (Lu et al., 2007; Roth et al., 2009). Loss of *Itgb4* in mice leads to severe blistering associated with Th2 inflammatory response, which suggests involvement of immune cells in EB pathophysiology (Han et al., 2018). Knockout of *Col7a1* recapitulates RDEB and creates a chronic wound condition underscoring its role in maintenance of epidermal-dermal integrity. Collagen-VII deficient wounds display suprabasal expression of α6β4 that lead to a dysregulated α6β4-laminin-332-JNK-STAT3. This, in turn, delays wound re-epithelialization and prolongs the presence of inflammatory CD11b cells in skin (Nyström et al., 2013). Interestingly, depletion of collagen-VII from immunodeficient mice (NSG mice) results in a less severe disease phenotype. Taken together, these studies emphasize the critical role played by the immune cells in exacerbating the disease condition (Esposito et al., 2016).

Blisters in the bullous diseases are usually associated with increased infiltration of cytotoxic T cells, B cells, neutrophils and other immune cells as well as their secreted cytokines (Hussein et al., 2007). Previous work has suggested that there is a substantial increase in the levels of cytokines such as IL-1, IL-6, IL-15, and TNF-α, and chemokines such as CXCL-1, CXCL-2, CXCL-3, CCL-5, CCL-27 in the blister fluids of EB patients compared to healthy controls. Moreover, the cytokine and chemokine expression profile varies between EB subtypes such that early DEB and JEB blisters expressed lower levels of cytokines and chemokines compared to that of early EBS (Alexeev et al., 2017). In EBA, several groups have shown that autoantibody production against COL7A1 is mediated through a milieu of neutrophils, macrophages, B cells and T cells and these phenotypes are ameliorated by the depletion of neutrophils and T cells (Sitaru et al., 2010; Iwata et al., 2013; Bieber et al., 2016).

MMPs secreted by immune cells can also influence the recruitment pattern of other immune cells to the injured tissue. Studies have shown that the severity of RDEB is a function of the type of MMP synthesized, its expression level and the balance between MMPs and TIMPs (Bodemer et al., 2003). Interestingly, a SNP (single nucleotide polymorphism) in the *MMP1* gene has also been implicated in increasing disease severity by influencing the expression of collagen-VII (Titeux et al., 2008). Likewise the correlation between MMP-9 and blister formation was shown in a study where treatment with doxycycline derivative, an inhibitor of MMP-9, alleviated the phenotype (Lettner et al., 2013). Collectively MMPs can serve as effective therapeutic targets.

Since DEB is a disorder of the basement membrane zone (BMZ), it is not surprising that, in RDEB patients that lack collagen-VII, two major BM components collage-IV and laminin-332 display a disrupted distribution pattern and do not co-localize at the BMZ (Onetti Muda et al., 1996). The disruption precedes blister formation suggesting that it could be a direct consequence of the lack of collagen-VII. Dermal-epidermal splitting also leads to an increase in the expression of TNC at the site of the split, which could be in response to the cytokines secreted either by epidermis or the recruited immune cells (Schenk et al., 1995). These studies suggest that TNC can serve as a potential link between the immune and ECM components in DEB.

Interaction between collagen-VII and cochlin is required to mount an immune cell mediated antibacterial response. In RDEB patients, absence of this interaction leads to increased bacterial colonization that further exacerbates the disease condition (Nyström et al., 2018). Lack of collagen-VII in RDEB destabilize the BMZ and cause the release of TGF-β associated with ECM increasing its bioavailability. TGF-β mediated activation of dermal fibroblasts, in turn, cause excessive deposition of ECM components (Nyström and Bruckner-Tuderman, 2018). In a recent case study, monozygotic twins suffering from RDEB exhibited distinct phenotypes due to changes in the expression of TGF-β target genes, despite having similar loss of collagen-VII. The more affected individual displayed increased fibrosis, which was ameliorated by treatment with the anti-fibrotic the matricellular protein decorin (Odorisio et al., 2014). These data point to the complexity of managing and treating RDEB, a genetic disease that is associated with loss in ECM organization which is further exacerbated by TGF-β mediated alteration in ECM dynamics. The involvement of matricellular proteins in mounting an immune response in EB provides an additional avenue for therapeutic intervention. Taken together, while EBs remain one of the most challenging diseases to manage and treat, and future treatment paradigms will need to take into account the immune-ECM interactions.

### Skin Cancers

Skin cancers are broadly classified as melanoma and non-melanoma types. Melanoma is an aggressive skin cancer that originates from melanocytes; the pigment producing cells in the skin (Villanueva and Herlyn, 2008). Non-melanoma skin cancers include Squamous cell carcinoma (SCC), Basal cell carcinoma (BCC), and Merkel cell carcinoma (MCC). SCCs originate from interfollicular stem or progenitor cell population in the epidermis and BCCs from the progenitor cells present in interfollicular epidermis and upper infundibulum (Donovan, 2009; Wang et al., 2011). MCCs, on the other hand, are the cancer of neuroendocrine (merkel) cells present in the basal layer of the epidermis (Munde et al., 2013). Other aggressive forms of skin cancer include cutaneous T and B lymphomas, a varied group of extranodal lymphoproliferative disorders associated with abnormal accumulation and proliferation of T and B lymphocytes in skin (Pandolfino et al., 2000; Siegel et al., 2012). Cutaneous T cell lymphomas are the most common with 65% incidence rate as compared to B cell lymphomas with a 25% incidence rate (Sokołowska-Wojdyło et al., 2015). All skin cancer types show a positive correlation with exposure to UV radiation as well as mutations in genes associated with tumor suppression, DNA repair, apoptosis and cell cycle regulation (D'Orazio et al., 2013; Kandoth et al., 2013).

Tumor resident and recruited immune cells play a dynamic role in regulating cancer progression. The tumor and its stroma recruit different populations of immune cells by releasing chemotactic factors and cytokines resulting in a chronic inflammatory condition (Binnewies et al., 2018). The immune cell repertoire, associated with the tumor microenvironment includes monocytes, macrophages, neutrophils, dendritic cells, natural killer cells, T and B cells (Cavallo et al., 2011). Factors such as CCL-2, CCL-5, CCL-17, CCL-22, CXCL-12, M-CSF, VEGF, and TGF-β synthesized by the tumor stroma aid in the recruitment of monocytes and subsets of T cells (Owen et al., 2003; Hiratsuka et al., 2006; Yang et al., 2008). In melanomas, the chemokines CCL-2, CCL-3, CCL-5, CXCL-9, and CXCL-10 recruit CD8^+^ T cells (Harlin et al., 2009). In a recent study it was shown that, tumor derived factors such as colony stimulating factor-1 (CSF-1), CCL-2, IL-34, and VEGF recruit circulating macrophages to the melanomas (Pieniazek et al., 2018). In human melanomas, CCL-2, also known as MCP-1, is associated with an increased inflammatory response and recruitment of macrophages and T cells (Ilkovitch and Lopez, 2008). Similarly in BCCs, CXCL-9, CXCL-10, and CXCL-11 recruit CXCR-3 expressing effector and memory T cells (Lo et al., 2011). In BCCs, the chemokines, CCL-17, CCL-18, and CCL-22 that recruit Tregs are overexpressed in the tumor islands and peritumoral skin. The stromal cells in turn synthesize CCL-17, which suggests a mechanism for the Treg infiltration seen in the stroma surrounding the BCC nests (Omland et al., 2016). In MCC, a recent study has shown that over expression of CCL-17 and its receptor CCR-4, aid in recruitment of CD4^+^ Tregs, Th2, and Th17 cells (Rasheed et al., 2018). Macrophage derived CCL-18 in cutaneous T cell lymphomas (CTCL) recruit T lymphocytes to the skin and is associated with enhanced invasion (Wu et al., 2014). Overall, the tumor and its stroma recruit a plethora of immune cells that contributes to tumor progression.

Dynamic crosstalk between the stroma and immune cells, change the tumor milieu from a tumor suppressive (or immunogenic) to tumorigenic (or immunosuppressive) state. Specifically, tumor associated macrophages (TAMs) switch from pro-inflammatory M1 macrophages, that are antigen presenting cells, to anti-inflammatory M2 macrophages, that are tissue remodeling and tumor growth promoting cells (Tariq et al., 2017). M1 macrophages phagocytose tumor cells, produce reactive oxygen (ROS) and nitrogen (RNS) species and secrete pro-inflammatory cytokines such as IL-6, IL-23, IL-12, and TNF-α that activate Th1 response (Komohara et al., 2014; Noy and Pollard, 2014). In contrast, M2 macrophages express anti-inflammatory cytokines like IL-10 and TGF-β that play a role in promoting tumor growth (Aras and Raza Zaidi, 2017). Depletion of M2 macrophages by treating with clodronate in CTCL xenograft mouse models, reduces angiogenesis and tumor growth (Wu et al., 2014). Human keratinocytes can inactivate T cell response by downregulating signaling pathways critical for T cell recruitment and cytotoxicity (Bronte and Zanovello, 2005; Bluth et al., 2009). In SCCs and BCCs, immunosuppression is mediated by preventing the recruitment of T cells to the cancer site through the activation of EGFR-Ras-MAPK signaling pathway (Pivarcsi et al., 2007). Interestingly, Tregs are known to suppress immune response by producing IL-10 and TGF-β and compete for T-cells-activating pro-inflammatory cytokines (Jarnicki et al., 2006). They are critical for inducing tolerance to self-antigens and also inhibit the antigen presenting ability of DCs (Sakaguchi et al., 2009). The transcription factor FOXP3 is important in regulating Treg function. Increased *FOXP3* expressing Tregs correlates with poor disease outcome in SCCs, BCCs and melanomas (Moreira et al., 2010). In BCCs, recruited Tregs build an immunosuppressive niche by locally synthesizing TGF-β (Omland et al., 2016). In MCC, M2 macrophages and Tregs present in the tumor maintain an immunosuppressive state contributing to a pro-tumorigenic microenvironment (Gaiser et al., 2018). Interestingly, increased recruitment of CD8^+^ T cells to the skin is associated with better prognosis in MCCs (Touzé et al., 2011).

Taken together, there is growing evidence that tumor microenvironment in cancers develop immune suppressive mechanisms to evade the T cell mediated cytotoxicity that can promote metastasis.

The ECM in cancer is highly remodeled and disorganized due to aberrant deposition by activated fibroblasts and enzymatic remodeling by the tumor and immune cells (Karagiannis et al., 2012). Changes in ECM dynamics promote tumor growth by regulating proliferation, survival, adhesion and migration (Pickup et al., 2014). The BM in melanomas has reduced thickness compared to healthy controls due to the absence of collagen-VII (Schmoeckel et al., 1989). A study of the BM in BCC affected skin showed aberrations in bullous pemphigoid antigens possibly due to its abnormal synthesis by the tumor associated cells (Stanley et al., 1982). Similarly, the BM structure in aggressive SCCs is irregular and discontinuous (Sakr et al., 1987). Matrix metalloproteinases such as MMP-1, -2, -3, -8, -9, -10, -13, -15, -16, and -26 can facilitate tumor growth, invasion, and angiogenesis in BCC, SCC and melanomas (Pittayapruek et al., 2016). In a recent study, expression profiling of BCCs showed an increase in ECM remodeling proteins including MMP-1, -3, -8, -9, and Cathepsin-K indicating the important role played by these enzymes in cancer progression (Ciazynska et al., 2018). In organ culture models of BCC the increased expression of MMP-2 and MMP-9 correlates with degradation of collagen-I and -IV at the tumor front (Goździalska et al., 2016). Likewise, the expression of ADAM-10, -12, and -17 was specifically observed in deep invasion area of BCC biopsies and correlated with the loss of collagen-XVII (Oh et al., 2009). The TAMs in BCCs induce COX-2 expression, which, in turn, results in a COX-2 dependent release of MMP-9, VEGF, and FGF that promotes tumor invasion (Tjiu et al., 2009). Interestingly, MMP-9 producing inflammatory macrophages and neutrophils are associated with BM degradation in BCC patients (Boyd et al., 2008).

Similarly, in SCCs the γ2 chain of laminin-332 is overexpressed in the invasive front (Hamasaki et al., 2011). In a DMBA (7,12-dimethylbenz-anthracene)-TPA (12-O-tetradecanoyl-phorbol-13-acetate) induced mouse model of SCCs, overexpression of collagen-XVII, integrin α6β4 and the γ2 chain of laminin, promoted tumorigenesis (Moilanen et al., 2017). Increased laminin-332 expression with high MMP-2 and MMP-14 activity is seen in invasive SCCs with poor prognosis (Marinkovich, 2007). Reduced expression of collagen-VII in SCCs enhances invasion, dysregulates epithelial differentiation and drives epithelial to mesenchymal transition (Martins et al., 2009). Loss of collagen-VII expression (in RDEB) results in an upregulation of TGF-β signaling and increased angiogenesis in SCCs underscoring the critical role played by collagen-VII in suppressing TGF-β signaling (Martins et al., 2016). Transgenic mice lacking MMP-9 reduced the tumor invasiveness in a mouse model of SCC whereas, transplanting MMP-9 producing bone marrow cells increased tumor invasiveness (Coussens et al., 2000). The cleaved oligosaccharides generated by heparinize digestion of heparin sulfate chains is shown to play an important role in tumor progression in BCCs and SCCs (Pinhal et al., 2016). The abundance and stiffness of collagen also play an important role in melanoma progression where it stimulates proliferation and differentiation in a YAP/PAX3/MITF dependent manner (Miskolczi et al., 2018). In melanoma and BCCs, cathepsin K expression is associated with a fibromucinous stroma around the tumor nests, indicative of ECM degradation (Ishida et al., 2013). Interestingly, the matricellular protein POSTN is associated with metastatic progression in melanoma by attenuating adhesion and promoting migration (Fukuda et al., 2015). Tumor fronts of aggressive BCCs and SCCs overexpress TNC (Schalkwijk et al., 1991; Dang et al., 2006). Likewise, CCN-1 and CCN-2 are overexpressed in keratinocytes of BCCs in a YAP dependent manner promoting cell proliferation and survival (Quan et al., 2014).

The crosstalk between immune cells and ECM is thought to be critical in driving skin cancer pathogenesis. In patient samples of SSCs, an increase in CD163^+^ TAMs is associated with enhanced expression of MMP-9 and MMP-11, which promotes angiogenesis and tumor growth. The same study also reports the activation of IL-4 dependent STAT-6 signaling in TAMs that induces differentiation of Th2 cells, further maintaining an immune-suppressive environment (Pettersen et al., 2011). In a DMBA-TPA induced mouse model of SCC, Gr1^+^ neutrophils produce remodeling enzymes including myeloperoxidase (MPO) and elastase, which are associated with poor disease outcome (Gasparoto et al., 2012). TNF-α and TGF-β secreted by macrophages synergistically increase melanoma migration in a NF-κB dependent manner through the release of MMP-1 and MT1-MMPs (Li R. et al., 2017). Hyaluronic and proteoglycan link protein (HAPLN-1) is an ECM protein highly expressed in young fibroblasts compared to aged fibroblasts and is important for maintaining stability of hyaluronan and collagen (Ecker et al., 2019). Since T cells use collagen fibrils to infiltrate tumors, downregulating HAPLN-1 expression in melanoma models results in decreased CD3^+^ T cell infiltration associated with increased tumor cell extravasation. On the other hand treatment with recombinant HAPLN-1 increases the T cell recruitment and reduces the tumor growth. The association between collagen fiber stability and T cell migration has been suggested as one of the reasons for aggressive melanomas in aged individuals (Kaur et al., 2018). In CTCL, T lymphocytes localize toward the epidermis by expressing integrin α3β1 allowing them to bind with laminin-332 and migrate through the BM, which interestingly does not result in ECM disorganization (Wayner et al., 1993). Tumor derived OPN binds to macrophages through integrin α9β1 resulting in the overexpression of COX-2 and MMP-9 and promotes angiogenesis in melanomas (Kale et al., 2014). In melanoma models, IFN-γ induced by TSP-1 overexpression is critical for macrophage recruitment and polarization to the M1 state (Martin-Manso et al., 2008). This points to an important role played by TSP-1 in suppressing tumor growth.

**Figure 3:**
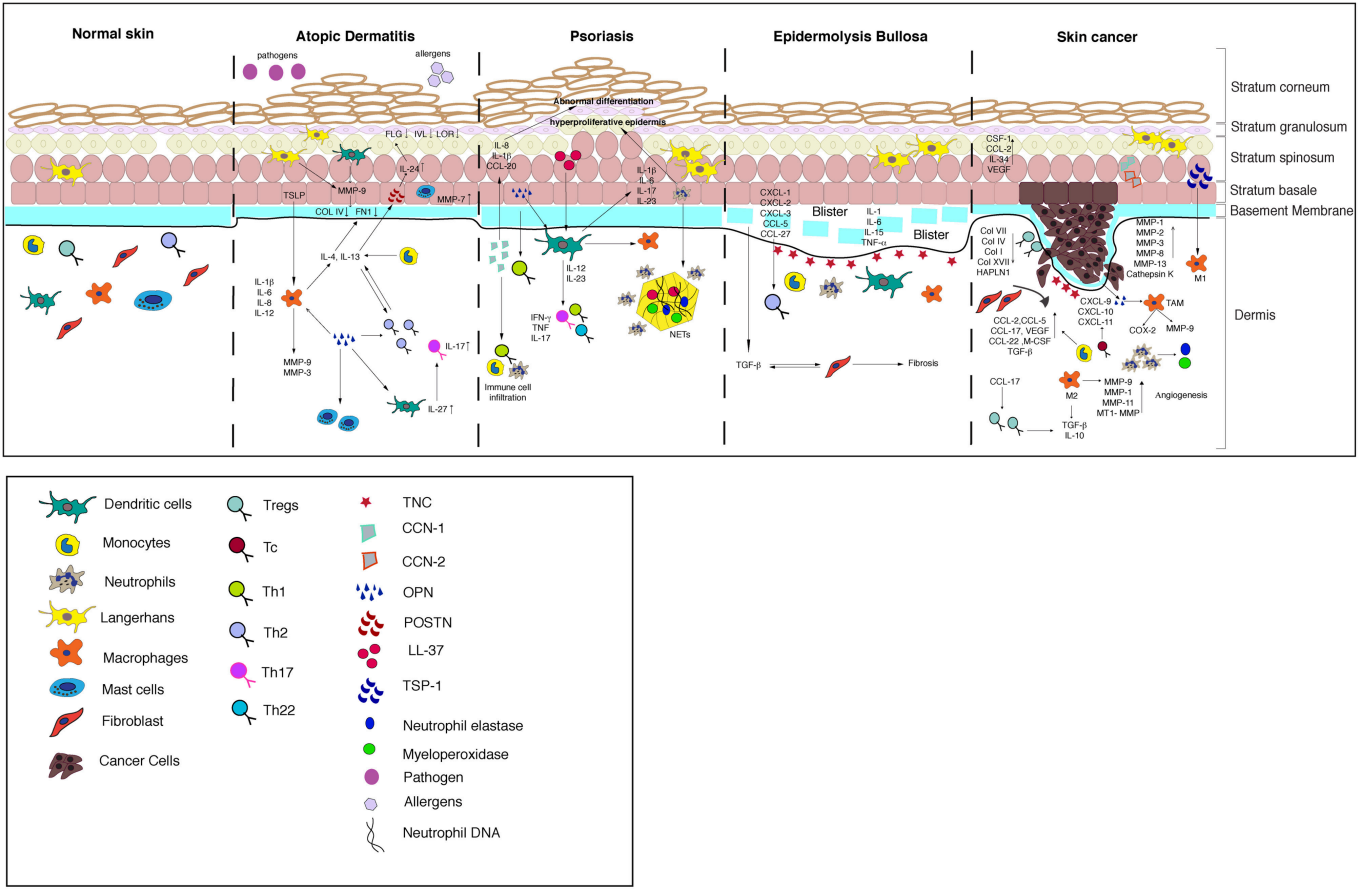
A model summarizing the crosstalk between the ECM and immune cells in the skin diseases discussed in this review.

The ECM-immune cell interactions have been well studied in the context of cancer since the ECM is highly remodeled by both the tumor and immune cells to facilitate the tumor growth. The tumor microenvironment can switch from being anti-tumorigenic to pro tumorigenic depending on dynamic interactions between the tumors; ECM and immune compartments and can result in aggressive cancer with poor prognosis. Understanding the signaling events regulating this process has led to the development of therapies targeting the immune system as evidenced by advent of immunotherapy in treating cancer. Figure 3 summarizes the ECM-immune interactions in all of the skin diseases discussed in this review.

## Current State of the Art Therapeutic Interventions

Currently available treatment approaches for skin diseases involve treatment with anti-inflammatory drugs, analgesics, topical steroids, and anti-histamines (Grassberger et al., 2004; Abraham and Roga, 2014). Conventional therapies for the treatment of the diseases discussed in this review are listed in the Table 3. With the advent of techniques such as nanomedicine, gene therapy (genetic engineering), stem cell transplantation, and cell and protein replacement therapy, curative treatment of skin diseases seem achievable and have been discussed below.

**Table 3:** Conventional treatment strategies.

**Skin Disease**	**Drugs**	**Mode of action**	**References**
**Atopic Dermatitis**	Calcineurin inhibitors: tacrolimus and pimecrolimus	Prevent the activation of NFAT by inhibition of calcineurin phosphatase resulting in prevention of activation of T cells, mast cells, and cytokines like IL-4, -5, - 31, TNF-α.	Mayba and Gooderham, 2017; Papier and Strowd, 2018
	Phosphodiesterase inhibitors: crisaborole and Apremilast.	Prevent the conversion of cAMP to AMP that leads to accumulation of cAMP. This in turn causes suppression of NFAT, and NFKB pathways involved in inflammation.	
	Janus Associated Kinase-Signal Transducer and Activator of Transcription (JAK - STAT) inhibitors: Tofacitinib	Inhibit phosphorylation of JAK-1 and JAK-3 and prevent the activation of STATs.Reduce immune cell polarization and cytokine production.	
	Cytokine and cytokine signaling inhibitors: Dipulimab, Lebrikizumab, and Nemolizumab.	Dipulimab is an antibody that binds to the alpha subunit of the IL-4 receptor. This inhibits the pathways driven by IL-4 and IL-13 and reduces the expression level of genes involved in epidermal hyperplasia and immune cell activation. Lebrikizumab is an antibody that blocks IL-13 and the signaling pathways associated with it. Nemolizumab is an antibody against receptor A of IL-31. It blocks homing of CLA^+^ T cells and decrease pruritus.	
	Systemic immunosuppressants: Ciclosporin A, Methotrexate, and Benvitimod	Inhibition of immune cell recruitment and production of pro-inflammatory cytokines.	
	Antihistamines: Hydroxyzine, Diphenhydramine, Chlorpheniramine	Inhibition of allergic responses and associated pruritis.	
**Psoriasis**	Vitamin D3 analogues: Calcitriol, Tacalcitol, and Calcipotriol.	Antiproliferative, pro-differentiation, suppression of T cell activation, increased production of Tregs, MHC-II suppression.	Duvic et al., 1997; Trémezaygues and Reichrath, 2011; Uva et al., 2012; Almutawa et al., 2013; Jacobi et al., 2015; Torsekar and Gautam, 2017
	Calcineurin inhibitors Pimecrolimus, and Tacrolimus.	Same as above.	
	Keratolytics - Salicyclic acid, and urea.	Increases shedding of corneocytes and reduction of pH.	
	Topical corticosteroids	Vasoconstrictive, antiproliferative, anti-inflammatory, and immunosuppressive.	
	Retinoids Tazarotene, and Acitercin	Binds to family of retinoic acid receptors - down-regulates keratinocyte differentiation, proliferation, and inflammation.	
	UV based therapy	Inhibition of inflammatory pathways, induction of apoptosis, downregulation of TH1/TH17 inflammatory axis, cell cycle arrest.	
	Phosphodiesterase inhibitor: Apremilast	Same as above	
	Systemic immunosuppressants: Ciclosporin, and Methotrexate.	Same as above	
	Cytokine and cytokine signaling inhibitors: Adalimumab, Etanercept, Infliximab, Secukinumab, and Ustekinumab.	Adalimumab, Etanercept, and infliximab are antibodies against TNF that result in inhibition of inflammation and immune cell recruitment. Secukinumab binds and neutralizes IL-17 that cause reduction in inflammation, immune cell recruitment and epidermal hyperproliferation. Ustekinumab binds to common receptor for cytokines IL-12 and IL-23 thereby reducing the activation and differentiation of Natural killer (NK) cells and CD4^+^ T cells.	
	Anti-hyperproliferative Dithranol	Impedes DNA replication and reduces elevated cGMP levels.	
**EB**	Treatment involves draining blisters using sterile needles, surgeries for separating fused digits.		Nystrom et al., 2015
	A small-molecule angiotensin II type 1 receptor antagonist Losartan	Reduction in TGFβ expression slowing down fibrosis in RDEB.	
	NSAIDs, analgesics	Suppress inflammation.	Goldschneider et al., 2014
**Squamous cell carcinoma**	Single or in combinations—Surgery, thermal ablation, radiation		Ribero et al., 2017; Yanagi et al., 2018
	Epidermal growth factor receptor (EGFR) inhibitor: Cetuximab, Panitumumab, gefitinib, and erlotinib	Cetuximab and Panitumumab monoclonal antibody binds EGFR and inhibits EGF mediated signaling. Gefitinib and Erlotinib bind to tyrosine kinase domain of EGFR.	
	Tyrosine kinase inhibitors: imatinib	Imatinib binds close to ATP binding site of tyrosine kinases thereby preventing its activity.	
	26S proteasome inhibitor: Bortezomib	Bortezomib contains a boron atom which binds to active site of 26S proteasome and prevents degradation of ubiquitin tagged proteins thereby enhancing cell death.	
	Apoptosis inducer: Isotrenoin	Induces apoptosis in sebaceous gland cells and enhances neutrophil gelatinase-associated lipocalin (NGAL) production which induces sebocyte apoptosis.	
	Cytotoxic drugs: 5-Fluorouracil (5-FU), Doxorubicin, and Cisplatin	5-FU is an inhibitor of thymidylate synthase, which inhibits DNA replication. Doxorubicin inhibits topoisomerase II action. Cisplatin cross links DNA and prevents mitosis.	
	Immune checkpoint inhibitors: Anti-PD-1: Nivolumab and Pembrolizumab Anti-CTLA-4: Ipilimumab	Cytotoxic T cell mediated killing of tumor cells.	
**Basal cell carcinoma**	Surgical excision, cryotherapy, Laser therapy, Mohs micrographic surgery, photodynamic therapy and radiotherapy.		Lewin and Carucci, 2015; Migden et al., 2018
	Inhibitors of DNA replication: 5-FU.	Same as above.	
	Hedgehog pathway inhibitors: vismodegib and sonidegib	It is an antagonist of smoothened which in turn causes inactivation of GLI-1 and GLI-2 transcription factors.	
	Immune response modifier: imiquimod	Activates innate immune cells and incites an inflammatory response. This, in turn, activates adaptive immune cells.	
**Melanoma**	BRAF inhibitor: vemurafenib and dabrafenib	Inhibit the mutated kinase domain of BRAF involved in MAPK signaling.	Johnson and Sosman, 2015
	MEK inhibitor: Trametinib	Allosteric inhibitor of MEK1/2 that is constitutively activated.	
	Immunotherapy: Interleukin-2	Activates immune system to attack cancer cells. Also stimulate melanoma cells to secrete chemoattractants for immune cells.	
	Anti-PD-1: Nivolumab and Pembrolizumab	Same as above.	
	Anti-CTLA-4: Ipilimumab	Same as above	
**Merkel cell carcinoma**	Mohs micrographic surgery, lymph node dissection and radiation therapy. Sentinel lymph node biopsy		Tello et al., 2018
	Chemotherapy: Somatostatin analogs	Inhibition of endocrine tumor growth.	
	mTOR inhibitors	Blocking PI3K/Akt/mTOR pathway and inhibit cell proliferation and tumor growth.	
	Immune checkpoint inhibitors:	
	Anti-PD-1: Nivolumab and Pembrolizumab avelumab	Same as above.	
	Anti-CTLA-4: Ipilimumab	Same as above.	
**Cutaneous lymphoma**	Topical Corticosteroids		Wollina, 2012
	Phototherapy	Use of UVA on skin, which results in self destruction of T cells.	
	Topical chemotherapy: Mechlorethamine and carmustine.	Chemically modify DNA and prevent tumor growth.	
	Topical imiquimod	Aid in the release of IFN-γ and cytokines for suppressing tumor growth.	
	Cytokine therapy: TNF-α	Stimulate immune system to target tumor cells.	

### Nanomedicine

Nanomedicine involves the use of particles within the nanometer range, for the detection and treatment of diseases. They are effective drug delivery agents for targeted therapy that enhance drug bioavailability and facilitate controlled drug release for long-term effect. Various nanoparticles as delivery agents for treatment of skin diseases include liposomes, solid lipid nanoparticles, polymeric micelles and nanospheres, dendrimers, nanotubes, quantum dots, gold particles etc. that has been reviewed previously (Dianzani et al., 2014). In mouse and rat models of AD and psoriasis, nanoparticle based delivery of drugs such as tacrolimus, corticosteroids, retinoids, methotrexate, and cyclosporine result in better outcomes (Palmer and DeLouise, 2016). Similarly, doxorubicin-loaded cationic solid lipid nanoparticles (DOX-SLN) have shown better drug penetration and inhibition of skin cancer tumor growth (Huber et al., 2015). In RDEB, localized delivery of gold nanoparticle coated with epigallocatechin-3-gallate (E3G) in SCC tumor site has been shown to abrogate the adverse effect of the drug in the liver (Manoukian et al., 2014). Additionally in EB, a novel nanocapsule based wound dressing technique has been developed. Upon infection, it releases antibiotics and undergoes a color change that indicates response to an active infection (Zhou et al., 2018). The promise of nanomedicine provides new therapeutic opportunities for treating skin disorders that have been refractory to treatment so far.

### Gene and Stem Cell Therapy

Gene therapy involves the introduction of corrected copy of genes into cells for treating genetic disorders (Anguela and High, 2019). Likewise, stem cell therapy refers to stem cell transplantation, either from the same (autologous) or from a different (allogeneic) individual replacing affected cells. Alternatively, gene-corrected autologous stem cells can be engineered to generate skin substitutes followed by transplantation (Karantalis et al., 2015). Gene and stem cell therapy have proven highly beneficial for genetic skin disorders such as EB which continues to be a major health burden due to lack of effective treatment. The retroviral mediated transfer of COL7A1 into fibroblasts and keratinocytes have shown successful incorporation of collagen-VII at the dermal-epidermal junction of the engineered skin grafts (Ferrari et al., 2006; Piaceski et al., 2018). In a recent path-breaking paper, physicians were able to repair the skin of a 7-year old JEB patient who had lost 80% of his epidermis. The JEB was caused by a mutation in the *LAMB3* gene. Autologous non-lesional keratinocytes were used to introduce the corrected gene copy, and these cells were used to generate epidermal skin grafts. The patient showed complete recovery 2 years after the skin was grafted (Hirsch et al., 2017).

Under homeostatic conditions, bone marrow derived mesenchymal stem cells have been shown to regulate proliferation and maturation of T cells, NK cells, B cells, and dendritic cells. However, this regulation is perturbed in AD and psoriatic patients and bone marrow transplantation has been shown to result in remission of diseases (Li et al., 2015; Shin et al., 2017). Similarly, transplantation of bone marrow derived mesenchymal stromal cells into RDEB patients showed symptomatic improvement (Uitto, 2008).

Genetically engineered bone marrow cells have been designed to carry tumor antigens for targeting skin cancers. Autologous transplantation of lymphocytes that are genetically modified to express tumor antigen MAGE-A3 has been shown to mount effective tumor killing response in melanoma (Fontana et al., 2009). Furthermore, engineering T cells with NY-ESO-1, melanoma antigen resulted in tumor regression in clinical trials (Dummer et al., 2008). Adenovirus mediated transduction of T cells with IL-2 has also shown improved prognosis in clinical trials (Puzanov and Flaherty, 2010).

While at its infancy, stem cell therapies provide the promise of a “cure” particularly for devastating skin blistering disorders. The challenge will be to reduce the costs of such treatments to make it more accessible to patients.

### Cell and Protein Replacement Therapy

Protein replacement therapy involves introduction of intact proteins for supplementing its loss from the patients and is primarily used in treatment of EB. Fibroblasts that can produce intact collagen-VII protein have been intradermally injected to RDEB patients and mouse models. The recipients, in turn, showed increased production of collagen-VII at the dermal-epidermal junction and enhanced wound healing (Nyström et al., 2013). Likewise, in mice that were grafted with skin from RDEB patients, topical application of recombinant collagen-VII protein to wounds resulted in collagen-VII incorporation in the wounded regions, and efficient repair (Uitto, 2008). More work will be required in the future to test the efficacy of this treatment in restoring normal skin function.

## Conclusion, Perspectives and Future Directions

In this review we have highlighted the signaling and regulatory cascades central to the interactions between ECM and immune cells in several skin diseases. Based on the extensive literature reviewed, it is conceivable that the pathways governing this crosstalk can serve in developing novel effective targets for therapeutic interventions. Therefore, an important area of future research would be to identify these cascades. While, in diseases like AD, Psoriasis, and cancer, the interaction is well understood, further investigation is required to discern the crosstalk in EB. Additionally, skin disease conditions are often accompanied by a systemic response thereby, necessitating the use of combinatorial therapies that target both the local and systemic responses. While available therapies primarily rely on mitigating inflammatory response, directly targeting of the ECM–immune cell pathways might contribute to better disease outcome. Additionally, with the emerging functions of matricellular proteins such as decorin, POSTN, OPN, and CCN-1 in driving the skin diseases, a thorough investigation of their roles could further aid in better understanding of disease pathophysiology. Ongoing state of art therapeutic interventions such as stem cell transplantation, immunotherapy, gene therapy, and nanomedicine hold the promise for treating the most intractable of these conditions.

## Author Contributions

All authors listed have made a substantial, direct and intellectual contribution to the work, and approved it for publication.

### Conflict of Interest Statement

The authors declare that the research was conducted in the absence of any commercial or financial relationships that could be construed as a potential conflict of interest.
